# Rebamipide, an Amino Acid Analog of 2(1H)-Quinolinone, Inhibits the Formation of Human Osteoclasts

**DOI:** 10.1155/2016/6824719

**Published:** 2016-11-14

**Authors:** Yuki Nanke, Tsuyoshi Kobashigawa, Toru Yago, Manabu Kawamoto, Hisashi Yamanaka, Shigeru Kotake

**Affiliations:** Institute of Rheumatology, Tokyo Women's Medical University, 10-22 Kawada-cho, Shinjuku-ku, Tokyo 162-0054, Japan

## Abstract

*Objectives*. Drug repositioning or drug reprofiling (DR) has recently been growing in importance. DR has a significant advantage over traditional drug development because the repositioned drug has already passed toxicity tests; its safety is known, and the risk of adverse toxicology is reduced. In the current study, we investigated the role of rebamipide, a mucosa-protecting agent, with recently reported anti-inflammatory function, in human osteoclastogenesis.* Methods.* Peripheral blood mononuclear cells (PBMCs) were cultured in the presence of M-CSF and sRANKL. Osteoclast formation was evaluated by immunohistological staining for CD51/61 (vitronectin receptors). Osteoclast formation, in the presence or absence of rebamipide (0, 1, and 3 mM), was observed by time-lapse photography and actin ring formation. The number of absorption sites and area of absorption were calculated using Osteologic™ plates. Pit formation was studied by 3D-SEM.* Results*. Rebamipide inhibited human osteoclast formation at 3 mM, a pharmacological concentration, and inhibited resorbing activity dose-dependently. Rebamipide induced the degradation of actin rings in mature osteoclasts. This mechanism may involve inhibiting the osteoclast fusion pathway through reducing the expression of DC-specific transmembrane protein (DC-STAMP).* Conclusions*. The present study suggests that rebamipide would be useful as a novel agent for osteoporosis and rheumatoid arthritis.

## 1. Introduction

Drug repositioning or drug reprofiling (DR) has recently been growing in importance [1]. DR has a significant advantage over traditional drug development because the repositioned drug has already passed toxicity tests; its safety is known, and the risk of adverse toxicology is reduced. Using DR, pharmaceutical companies have achieved a number of successes. The use of repurposed drugs can bypass much of the early cost and time needed to bring a drug to market. At this point, to investigate new pleiotropic effects of drugs is very meaningful. For example, Mundy et al. reported that the statins, drugs widely used for lowering serum cholesterol, also enhance new bone formation, and thus statins may be used for the treatment of osteoporosis [2].

Rheumatoid arthritis (RA) is a chronic inflammatory disease characterized by synovitis and the destruction of articular cartilage and bone [3, 4]. RA causes osteoporosis. Osteoclasts, multinucleated giant cells, contain calcitonin receptors and have the ability to form resorption pits on bone or dentin slices. Receptor activator of nuclear factor-*κ*B ligand (RANKL) induces osteoclastogenesis by binding to RANK. Osteoprotegerin (OPG) is a soluble decoy receptor of RANKL. These three molecules, OPG, RANK, and RANKL, play an important role in regulating osteoclastogenesis. Osteoclasts play a crucial role in both osteoporosis and bone destruction [5]. Thus, they are target cells to treat both RA and osteoporosis. In fact, some agents, such as anti-RANKL antibody, denosumab, which inhibits osteoclastogenesis, are now used as a drug for osteoporosis and RA.

We recently demonstrated that geranylgeranylacetone (GGA), a nontoxic inducer of heat shock protein, used as an antiulcer drug, potently inhibits human osteoclastogenesis induced by soluble RANKL [6] and also induces cell death in fibroblast-like synoviocytes (FLS) from patients with RA by inhibiting protein geranylgeranylation [7]. We showed the pleiotropic effects of GGA: antiosteoclastogenesis and anti-inflammatory effects.

Rebamipide (2-(4-chlorobenzoylamino)-3-[2(1H)-quinolinon-4-yl]propionic acid; OPC-12759, molecular weight: 370.79) is a mucosal protective agent used for the treatment of gastritis and gastric ulcer.Rebamipide is derived from a gastric mucosal stabilizer and gastric mucosal prostaglandin inducer. It was shown to increase the PGE_2_ content in the gastric mucosa and exhibit a gastric cytoprotective effect to prevent mucosal injury [8, 9]. This drug also directly scavenged hydroxyl radicals, suppressed superoxide production, and prevented inflammatory cell infiltration [10]. In addition, it inhibited the activation of NF-*κ*B, expression of interleukin-8 mRNA, and production of interleukin-8 in epithelial cells [11]. Also, it was recently reported that rebamipide works as an anti-inflammatory agent in both acute and chronic inflammation and has an inhibitory effect on proinflammatory cytokines [12].

In the current study, we investigated the effect of rebamipide, an amino acid analog of 2(1H)-quinolinone, on human osteoclastogenesis.

## 2. Materials and Methods

### 2.1. Chemicals and Reagents

Rebamipide was supplied by Otsuka Co., Ltd. (Tokyo, Japan). Recombinant human macrophage colony-stimulating factor (M-CSF) was obtained from Kyowa Hakko Kirin (Tokyo, Japan). Recombinant human soluble RANKL (sRANKL) was purchased from PeproTech (London, UK). For* in vitro* experiments, these reagents were dissolved in double-distilled water and adjusted to appropriate concentrations for each experiment.

### 2.2. Cells and Cell Culture for Osteoclastogenesis

Human peripheral blood was collected from healthy normal volunteers. Peripheral blood mononuclear cells (PBMCs) were isolated by centrifugation over Histopaque 1077 (Sigma, St. Louis, MO, USA) density gradients, washed, and resuspended at 1.3 × 10^6^ cells/mL in a-minimal essential medium (Gibco BRL, Gaithersburg, MD, USA) supplemented with 10% fetal bovine serum (JRH Biosciences, Lenexa, KS). PBMCs were then cultured for 3 days in 48-well plates in the presence of M-CSF (100 ng/mL). Nonadherent cells were then removed. Adherent PBMCs were cultured in the presence of various concentrations of rebamipide with human sRANKL (30 ng/mL). We used adherent PBMCs as monocytes in the culture system. Osteoclast formation was evaluated by immunohistological staining for CD51/61 (vitronectin receptors) after culture in the presence of sRANKL and M-CSF for 7 days. We identified vitronectin-receptor positive cells including more than three nuclei as osteoclasts.

### 2.3. Cell Proliferation Assay

Cell proliferation was measured using a Cell Proliferation Assay Kit (XTT baser) (Biological Industries Ltd., Israel). The assay was performed according to the manufacturer's protocol. Cells (3 × 10^5^/well) were cultured in 96-well plates. Before cells reached confluence, we cultured PBMC in the presence of M-CSF (100 ng/mL) and RANKL (30 ng/mL) and rebamipide (0, 1, and 3 mM). After 72 hours, the cells were collected, and cell proliferation was measured using the kit.

### 2.4. Human Osteoclast Formation (Time-Lapse Photography)

We cultured human osteoclasts in the presence of M-CSF (100 ng/mL) and RANKL (30 ng/mL). Osteoclast formation, in the presence or absence of rebamipide (0, 1, and 3 mM), was determined by time-lapse photography every one hour for 9 days.

### 2.5. Activation of Human Osteoclasts (Osteologic)

We cultured human osteoclasts in the presence of M-CSF (100 ng/mL) and RANKL (30 ng/mL) using Osteologic plates. Number of absorption instances and area of absorption were calculated.

### 2.6. Cellular Staining and Actin Structure

Mature osteoclasts induced by sRANKL as described above were cultured in the presence or absence of rebamipide (0, 1, and 3 mM) for 2 days. Osteoclasts were fixed with 10% formaldehyde in phosphate-buffered saline (PBS) for 10 minutes at room temperature and rinsed with PBS. Cells were then washed with Triton-X (0.1%) for 1 minute and stained with 20 U/mL rhodamine-phalloidin (Molecular Probes, Eugene, OR) in PBS for 30 minutes at room temperature.

### 2.7. Activation of Human Osteoclasts (Dentin Slices)

Human osteoclasts were cultured on dentin slices, and the absorption area was observed using SEM (JSM.6610LA, JEOL Ltd., Tokyo, Japan). Also, the absorption formation was assessed using 3D-SEM. Mature osteoclasts induced by sRANKL, as described above, were cultured in the presence or absence of rebamipide (0, 1, and 3 mM) for 2 days.

### 2.8. RNA Isolation and Quantitative Real-Time PCR (qPCR)

Total RNA was extracted from human CD14+ cells using the NucleoSpin® RNA kit (MACHEREY-NAGEL, Düren, Germany).

First-strand cDNA was synthesized from total RNA by reverse transcription using the ReverTra Ace® qPCR RT Kit (Toyobo, Osaka, Japan) according to the manufacturer's instructions.

Each cDNA sample was analyzed for gene expression by quantitative real-time PCR (qPCR) using the fluorescent TaqMan 5′-nuclease assay with an Applied Biosystems 7900HT sequence detection system (Applied Biosystems, Foster City, CA, USA). The TaqMan real-time PCR was performed using 2x TaqMan Master Mix and 20x premade TaqMan gene expression assays (Applied Biosystems). Glyceraldehyde-3-phosphate dehydrogenase (GADPH) was used as an internal standard of mRNA expression. Analysis was performed with ABI 7900HT SDS 2.2 Software and relative RNA quantification using the comparative Ct method, as described by Livak and Schmittgen [13].

Analysis of mRNA of integrin alpha V (ITGAV), integrin beta 3 (ITGB3), nuclear factor of activated T-cells cytoplasmic calcineurin-dependent 1 (NFATc1), T-cell leukemia translocation-associated gene (TCTA), voltage-dependent anion channel 1 (VDAC1), FBJ murine osteosarcoma viral oncogene homolog (FOS), tumor necrosis factor (TNFA), RANK, Jun protooncogene (JUN) DC-specific transmembrane protein (DC-STAMP), and colony-stimulating factor 1 receptor (CSF*α*R) on adding rebamipide was carried out.

To investigate the molecular mechanism of the effect of rebamipide on human osteoclastogenesis, mature human osteoclasts were cultured with rebamipide, and mRNA of ITGAV, ITGB3, NFATc1, TCTA, VDAC1, FOS, TNFA, RANK, JUN, DC-STAMP, and CSF*α*R was investigated.

### 2.9. Statistical Analysis

Data were analyzed with the Mann–Whitney test.

This study was approved by the ethics committee of our institution and carried out in compliance with the Helsinki Declaration.

## 3. Results

(1) Rebamipide inhibited human osteoclast formation (48-well plate).

**Figure 1:**
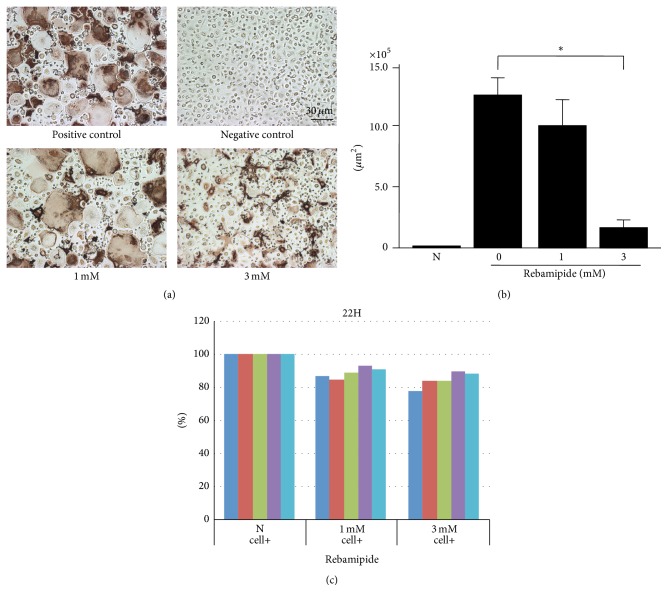
(a) Effect of rebamipide on human osteoclastogenesis. Rebamipide (0, 1, and 3 mM) dose-dependently inhibited the formation of human osteoclasts induced in the presence of sRANKL (30 ng/mL) and M-CSF (100 ng/mL). Immunohistological staining for CD51/61 (vitronectin receptors). (b) Effect of rebamipide on human osteoclastogenesis. ^*∗*^ *p* = 0.048. (c) XTT assay. The *y*-axis represents % expression of XTT assay compared with N (negative control without adding rebamipide). Each colored bar represents each level of normal volunteers.

Rebamipide (0, 1, and 3 mM) dose-dependently inhibited the formation of human osteoclasts induced in the presence of sRANKL (30 ng/mL) and M-CSF (100 ng/mL) (Figures 1(a) and 1(b)). The effect of rebamipide was not a result of cytotoxicity, because the concentrations were pharmacological [14].

(2) Rebamipide did not significantly inhibit the proliferation of cells although the cell proliferation was slightly inhibited at 1 and 3 mM (Figure 1(c)).

(3) Rebamipide inhibited human osteoclast formation (time-lapse photography).

Rebamipide inhibited human osteoclast formation at 3 mM, a pharmacological concentration, from the early phase of preosteoclasts (data not shown).

(4) Rebamipide inhibited human osteoclast formation (Osteologic plates).

**Figure 2:**
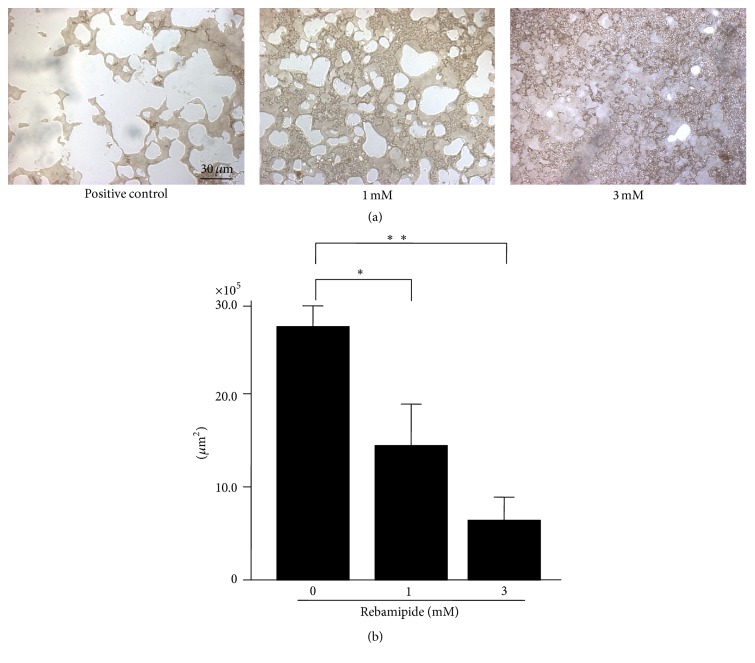
(a) Rebamipide inhibits human osteoclast formation (Osteologic). Rebamipide inhibits resorbing activity dose-dependently. (b) Effect of rebamipide on bone resorption by human osteoclastogenesis* in vitro* (Osteologic). ^*∗*^ *p* < 0.001; ^*∗∗*^ *p* = 0.0013.

Rebamipide inhibited the resorbing activity dose-dependently (Figures 2(a) and 2(b)).

(5) Rebamipide degenerates the formation of actin rings of mature osteoclasts.

**Figure 3:**
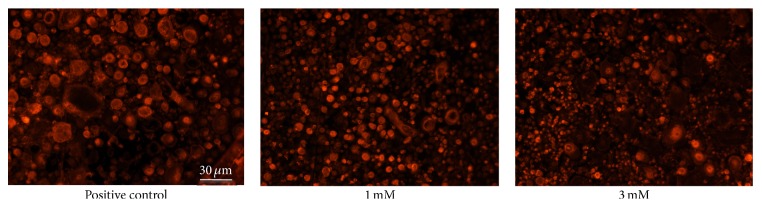
Rebamipide degenerates the formation of actin rings of mature osteoclasts. The formation of actin rings of a part of the mature osteoclasts was generated by adding rebamipide dose-dependently.

The degradation of actin rings as a part of mature osteoclasts was promoted by adding rebamipide dose-dependently (Figure 3).

(6) Human osteoclasts (dentin slice) were inactivated.

**Figure 4:**
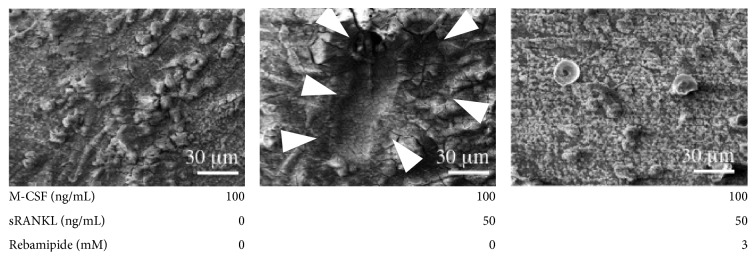
Activation of human osteoclasts (dentin slices). Middle panel: pit formation was seen on dentin slices by adding the positive control. Right panel: resorption pits on dentin slices were completely inhibited by adding rebamipide (3 mM).

Pit formation was seen on dentin slices in positive control. Resorption pits on dentin were completely inhibited by adding rebamipide (3 mM) (Figure 4). We used 3D-SEM to observe a single pit of each osteoclast. It is impossible to observe a low power view using 3D-SEM.

(7) Rebamipide reduced the expression of DC-STAMP mRNA.

**Figure 5:**
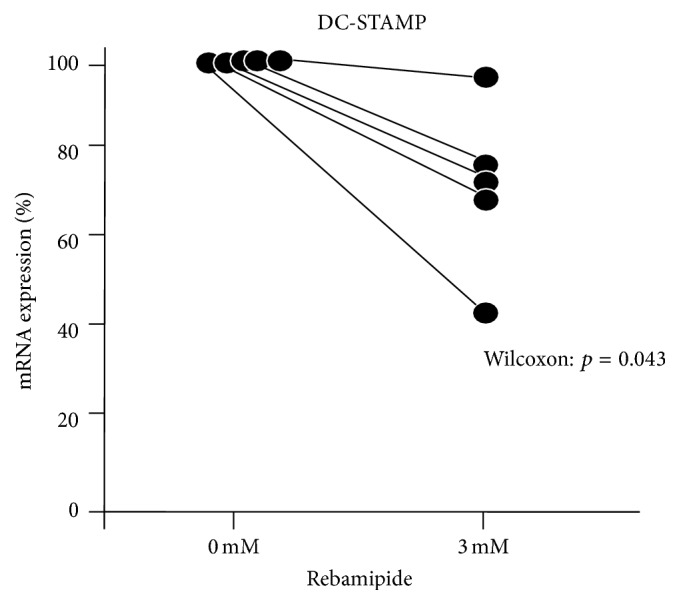
Rebamipide reduced the expression of DC-STAMP mRNA. The expression of DC-STAMP mRNA was reduced by adding rebamipide (3 mM) (*n* = 5).

The expression of only DC-STAMP mRNA was reduced by adding rebamipide (3 mM) (*n* = 5) (Figure 5).

## 4. Discussion

In the present study, we demonstrated that rebamipide dose-dependently inhibited human osteoclastogenesis induced by sRANKL. Rebamipide inhibited human osteoclast formation at pharmacological concentrations (3 mM) and inhibited resorbing activity dose-dependently. In addition, it induced the degradation of actin rings in mature osteoclasts at a pharmacological concentration. Thus, rebamipide has inhibitory effects on the formation and function of osteoclasts.

Rebamipide directly inhibited osteoclastogenesis from human monocytes since we used human monocytes alone from peripheral blood in our culture system. In addition, it reduced the expression of DC-STAMP mRNA; thus, these effects are suggested to inhibit the function of DC-STAMP in the osteoclast fusion pathway.

DR has become very important over the last few years [1]. DR has a significant advantage over traditional drug development because the repositioned drug has already passed toxicity tests; its safety is known, and the risk of adverse toxicology is reduced. Using DR, pharmaceutical companies have achieved a number of successes. The use of repurposed drugs can bypass much of the early cost and time needed to bring a drug to market. Thus, to investigate novel pleiotropic effects of drugs is very meaningful. We recently demonstrated the novel pleiotropic activity of GGA, a nontoxic inducer of heat shock protein, as an anti-gastric-ulcer drug [6, 7].

GGA potently inhibits human osteoclastogenesis induced by soluble RANKL [6] and induced cell death in FLS from patients with RA by inhibiting protein geranylgeranylation [7]. In this study, we have shown a novel pleiotropic effect of rebamipide on human osteoclastogenesis.

Rebamipide is a mucosal protective agent used for the treatment of gastritis and gastric ulcer. It is an optically active *α*-amino acid derivative of 2(1H)-quinolinone. The precise mechanisms are still unknown; however, it has a beneficial effect on the gastric mucosa through several mechanisms: increased gastric mucosal prostaglandin production [8], increased gastric mucus production [15], scavenging of hydroxyl radicals [16], inhibition of neutrophil activation [10], and suppression of gastric mucosal inflammation [17].

In addition to these effects, some other pharmacological actions of rebamipide have been reported, such as the regulation of apoptosis-related genes [18], inhibition of tyrosine nitration [19], promotion of Sonic hedgehog (Shh) restoration [20], correcting MAPK signaling abnormalities, counteracting the downregulation of *β*EGF expression, inhibiting the expression of apoptosis-related genes, and maintaining tight junctional complexes [21].

It was recently reported that rebamipide works as an anti-inflammatory agent in acute and chronic inflammation and it inhibited proinflammatory cytokines [12]. Experimental data have shown the protective effect of rebamipide on the intestinal barrier and its immunoregulatory properties. It is also capable of regulating lymphocyte proliferation and cytokine secretion and is expected to provide a new strategy for the treatment of ulcerative colitis patients [12].

As mentioned above, rebamipide scavenged hydroxyl radicals directly and suppressed superoxide production by polymorphonuclear leukocytes and prevented inflammatory cell infiltration.In addition to these pleiotropic pharmacological actions, rebamipide suppressed inflammatory cytokines. Choe et al. [22] reported that rebamipide inhibits tumor necrosis factor-*α*-induced interleukin-8 expression by suppressing the NF-*κ*B signal pathway in human umbilical vein endothelial cells. Kim et al. [23] reported that rebamipide inhibits endothelial adhesion to hypoxia/reoxygenation-stimulated endothelial cells via the NF-*κ*B-dependent pathway.

Some reports have demonstrated that rebamipide is effective for autoimmune disorders. Kohashi et al. [24] demonstrated that the administration of rebamipide effectively inhibits the autoimmune pathology in the NFS/sld mouse model of Sjögren's syndrome (SS). Rebamipide treatment suppressed the activation of CD4+ T-cells and Th1 cytokines (interleukin-2, interferon-*γ*), associated with impaired NF-*κ*B activity, and inhibited the expression of IRF-4B, a transcription factor associated with B-cell activation and differentiation. Kohashi et al. identified decreased apoptosis of salivary gland epithelia in rebamipide-treated mice. Since rebamipide inhibited the production of serum autoantibodies, IgM and IgG1, and induced a reduction in the transcriptional activity of IRF-4 via the downregulation of NF-*κ*B, rebamipide may represent a novel therapeutic approach for SS.

RA causes the inflammation of synovial membranes, produces IL-17 and RANKL [3], which infiltrate synovial membranes, and is involved in the formation of osteoclasts. T-cells activate macrophages and synovial cells and produce inflammatory cytokines, such as IL-1*β*, IL-6, and TNF-*α*. Their stimulation promotes the expression of RANKL in synovial cells and osteoblasts, thereby inducing osteoclastic differentiation [5]. Since rebamipide treatment suppressed the activation of CD4+ T-cells, rebamipide may be useful to treat bone destruction caused by osteoclasts. Recently, Moon et al. [25] reported that rebamipide suppresses collagen-induced arthritis through reciprocal regulation of Th17/Treg cell differentiation and heme oxygenase 1 induction. In addition, Byun et al. [26] reported that rebamipide attenuates autoimmune arthritis severity in SKG mice via regulation of B-cell and antibody production.

The concentrations of rebamipide (1–3 mM) used in the current study are within the range of pharmacology levels. It has been reported that a mucosal concentration of rebamipide is more than 0.2 mM after the oral intake of 10 mg/kg rebamipide in rats [27]. In addition, Suzuki et al. [28] reported that rebamipide (1 mM) attenuates* Helicobacter pylori*-induced gastric mucosal cell injury associated with neutrophil derived oxidants. It is also speculated that the concentration of rebamipide reaches 3 mM in the microenvironment* in vivo.* Thus, we performed our experiments using 0, 1, and 3 mM of rebamipide.

In summary, this study clearly demonstrated that rebamipide inhibited human osteoclastogenesis and induced the degradation of actin rings in mature osteoclasts. At least in part, this mechanism is due to inhibiting the osteoclast fusion pathway through reducing the expression of DC-STAMP. Next, we will examine whether the inhibitory effects of rebamipide on osteoclastogenesis* in vitro* are accompanied by inhibitory effects on bone loss. In this study, we showed another pleiotropic effect of rebamipide. The findings suggest that rebamipide may represent a novel therapeutic approach for osteoporosis and RA.
